# Pathophysiology of endothelial dysfunction in Fontan circulation: from bench to bedside and back again

**DOI:** 10.1007/s10456-025-09996-2

**Published:** 2025-08-21

**Authors:** Amalia Baroutidou, Artemios G. Karagiannidis, Theodoros Dimitroulas, Vasileios Kamperidis, Antonios Ziakas, Konstantinos Dimopoulos, George Giannakoulas

**Affiliations:** 1 First Department of Cardiology, AHEPA University Hospital, Aristotle University of Thessaloniki, St. Kiriakidi 1, 54636 Thessaloniki, Greece; 2 First Department of Nephrology, Hippokration Hospital, Aristotle University of Thessaloniki, Thessaloniki, Greece; 3 Fourth Department of Internal Medicine, Hippokration Hospital, Aristotle University of Thessaloniki, Thessaloniki, Greece; 4 Adult Congenital Heart Centre and Centre for Pulmonary Hypertension, Royal Brompton Hospital, Royal Brompton and Harefield Hospitals, Guy’s and St Thomas’ NHS Foundation Trust, Sydney Street, London, SW3 6NP UK; 5 National Heart and Lung Institute, Imperial College London, London, UK

**Keywords:** Endothelial dysfunction, Vascular dysfunction, Pathophysiology, Fontan, Single ventricle, Univentricular heart

## Abstract

The Fontan procedure is a definitive surgical approach for complex cardiac malformations, redirecting systemic venous blood into the pulmonary circulation through a staged repair that separates systemic and pulmonary venous returns in the absence of a subpulmonary ventricle. The ensuing unique hemodynamic conditions compromise the endothelial function both in the pulmonary and the systemic circulation. The underlying pathophysiological mechanisms, although distinct within each vascular bed, are interrelated and may collectively contribute to progressive end-organ dysfunction, ultimately accounting for the significant morbidity burden in Fontan patients. This review provides an overview of the current knowledge on the pathophysiology of pulmonary and systemic vasculopathy in Fontan circulation, with particular emphasis on the interplay between endothelial dysfunction and adverse clinical outcomes. Remaining gaps in knowledge and directions of future research are also discussed.

**Graphical abstract:**

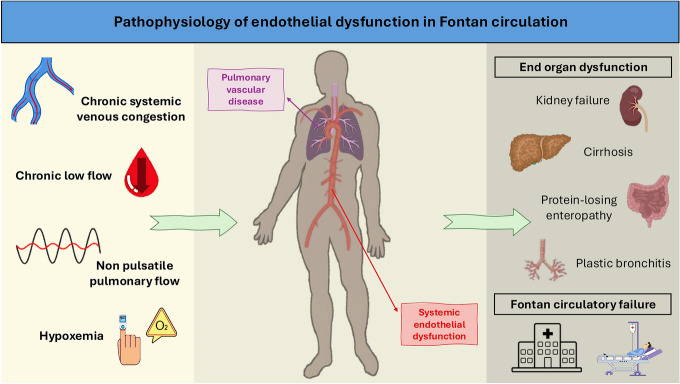

## Introduction

The univentricular heart encompasses a diverse group of congenital heart defects characterized by the absence or hypoplasia of either the right or left ventricle, rendering biventricular repair unfeasible [1]. Τhe Fontan procedure is the definite treatment for such complex cardiac malformations and involves redirection of systemic venous blood directly into the pulmonary circulation [2]. It is a staged surgical palliation that separates the systemic and pulmonary venous returns, in the absence of a subpulmonary ventricle, effectively re-establishing their connection in series [2]. Since its inception, several modifications have been implemented to the original procedure, aimed at optimizing the flow of systemic venous return to the pulmonary arteries [2]. With the advent of Fontan surgery, the prognosis of patients with single ventricle has improved substantially, with survival rates surpassing 80% 30 years after the operation [3, 4].

Despite advances in treatment, long-term complications (e.g. ventricular dysfunction and heart failure, chronic venous insufficiency, arrhythmias) progressively arise and morbidity remains significant [2]. Multiple factors contribute to Fontan circulatory failure including altered pulmonary hemodynamics, chronic upstream systemic venous congestion and reduced systemic ventricular preload due to the absence of a subpulmonary pumping ventricle [2]. Nevertheless, the exact mechanisms leading to hemodynamic compromise are still not completely elucidated. Emerging data indicate an intricate interaction between cardiac function and, both pulmonary and peripheral vasculature in patients following the Fontan procedure [5]. Endothelial dysfunction has been implicated as a potential contributing factor to long-term cardiovascular events [6, 7] leading to Fontan failure, and may serve as an early indicator of impaired functional status in this population [5, 8–10]. However, evidence on macro- and microangiopathy in the Fontan circulation has only recently begun to accumulate [7].

The current review presents the key pathophysiological mechanisms underlying endothelial dysfunction in both the pulmonary and systemic vascular beds in patients with a Fontan circulation. It also explores potential correlations between vascular injury and adverse clinical outcomes and provides guidance for future research in this population.

## The Fontan principle

The Fontan procedure significantly alters the physiology of the cardiovascular system, creating a unique circulation characterized by several hemodynamic derangements per se [11]. A neoportal system is established, as blood from the systemic capillary bed is directed to the pulmonary capillaries without the interposition of a cardiac pump [11]. Τhis pulmonary neoportal system acts as a dam between systemic venous return and the systemic ventricle, and serves as the key determinant of upstream venous congestion and downstream flow; therefore, the pulmonary circulation is regarded as the critical bottleneck in the Fontan circulation [11, 12].

Flow through this “bottleneck” is regulated by three elements: resistance caused by the “bottleneck” (pulmonary vascular resistance), upstream pressure (central venous pressure) and downstream pressure (atrial pressure, ventricular filling pressure) [12]. In the absence of a sub-pulmonary ventricular pump, central venous pressure (CVP) is the main driving force for pulmonary blood flow, with negative intrathoracic pressure during inspiration playing a supportive role in enhancing venous return [13]. With increasing impedance to flow secondary to increased ventricular filling pressures and pulmonary vascular resistance (PVR), CVP inevitably rises maintaining the transpulmonary gradient. However, there is a limit to how much CVP can rise, and pressures exceeding 18–20 mmHg are generally poorly tolerated [14]. Subsequently, PVR and the pulmonary vasculature in general remain to be the principal factor affecting cardiac output in the Fontan circulation [11, 12]. Indeed, even slight increases in PVR can cause pronounced fluctuations in cardiac output and systemic venous pressure [11, 13], ultimately leading to progressive end-organ dysfunction (e.g., cirrhosis, lymphatic dysfunction, protein-losing enteropathy, plastic bronchitis, venous thromboses, ascites, peripheral oedema, renal failure) and Fontan circulatory failure [11, 13].

## Endothelial dysfunction and pulmonary vascular disease in the Fontan circulation

### Abnormal pulmonary vascular development

In patients with single ventricle physiology, the pulmonary vascular bed is exposed to conditions that can influence its development during the early years of life, well before the Fontan procedure [13]. Palliative systemic-to-pulmonary shunts, commonly used in this population to augment pulmonary blood flow and allow the pulmonary arteries to develop, may also induce adverse vascular remodeling by excessive pulmonary flow and pressure (shear stress) [13, 15]. Inversely, reduced pulmonary blood flow, e.g., in cases of pulmonary stenosis or atresia, may lead to a diminished pulmonary vascular cross-sectional area and, consequently, increased PVR [13, 15]. In both cases, pulmonary vascular disease (PVD) may develop well before reaching the final stage of Fontan palliation [11, 13, 15].

### Non-pulsatile pulmonary flow and shear stress

The Fontan procedure introduces additional non- physiological characteristics to the pulmonary vascular bed, further affecting its structure and function [11, 13]. The attenuation or absence of pulsative flow into the pulmonary vasculature due to the lack of a subpulmonary ventricle is deemed to be the main mechanism involved in adverse pulmonary vascular remodeling in this population [16]. Experimental studies have shown that pulsatile flow plays a crucial role in maintaining a low PVR, through the passive recruitment of capillaries in pulmonary microcirculation, and by promoting the release of nitric oxide (NO) through shear stress, facilitating endothelium-dependent relaxation [17]. The effects of pulsatile shear stress on endothelium activation and NO release are mediated by the upregulation of endothelial nitric oxide synthetase (eNOS), through various complex mechanisms. Molecular pathways behind eNOS activation include rapid ion channel activation and eNOS protein phosphorylation by the serine/threonine protein kinase B (Akt), stimulated by the phosphoinositide 3-OH kinase [18]. Moreover, in vitro data suggest that pulsatile shear stress enhances the eNOS gene increasing NOS3 expression that regulates NO production [8].

The prolonged exposure to non-pulsatile pulmonary flow following the Fontan procedure reduces shear stress on the pulmonary endothelium, downregulating eNOS synthesis and activation, thereby diminishing NO-mediated endothelial-dependent vasodilation and increasing intimal fibrosis and apoptosis of vascular smooth muscle cells [16, 19]. This disruption in pulmonary endothelial function contributes to progressive adverse pulmonary vascular remodeling that elevates PVR [16, 19]. The hypothesis of impaired endothelial-dependent vasodilation in patients after Glenn or Fontan operation has been confirmed by small studies that demonstrated an attenuated pulmonary vasodilator response to acetylcholine (Ach), an endothelium-dependent vasodilator [20–22]. Response to nitroglycerin, an endothelium-independent vasodilator and exogenous NO donor, was however, preserved [20, 21]. This suggests that pulmonary endothelium-independent vasodilation, and, subsequently, pulmonary artery smooth muscle sensitivity to exogenous agents releasing NO remains intact [20, 21].

Pulmonary arterial hypertension (PAH) therapies have been used in this setting to reduce PVR and improve exercise capacity and quality of life [23]. Many studies, including randomized trials, have explored the efficacy of pulmonary vasodilators in selected patients with Fontan circulation, with some of them reporting slight yet significant improvements in hemodynamic parameters and exercise capacity [24–30]. A recent meta-analysis demonstrated that PAH-targeted therapies improved submaximal effort indices at the anaerobic threshold but had no effect on maximal effort, raising ongoing debate about the optimal indications and timing for their use in the Fontan population [31]. PAH therapies are aimed primarily at pulmonary vasoconstriction; nevertheless, PVD in Fontan patients appears multifactorial and is generated by more complex mechanisms than in other populations, a fact that might justify the suboptimal response of these patients to PAH pharmacological treatment. In fact, endothelial dysfunction may play a greater role in this perplexed scenario and could constitute a target for therapies in itself.

### Pulmonary arteriovenous malformations

Pulmonary arteriovenous malformations (PAVMs) have also been implicated in the pathogenesis of PVD in patients with single ventricle-physiology and a Fontan operation. PAVMs are structurally abnormal vessels that may be the result of incomplete development of the pulmonary capillary network. They allow shunting of desaturated blood from the pulmonary arteries to the pulmonary veins, bypassing the pulmonary capillary bed and alveoli where gas exchange occurs [32]. PAVMs can be present in normal lungs, but may proliferate and dilate under specific physiological conditions, becoming clinically significant with severe arterial oxygen desaturation [32].

The development of PAVMs in patients with Glenn shunts in the ipsilateral lung was initially attributed to excessive perfusion of the lower lobe (the predominant site of PAVM formation) or the absence of pulmonary pulsatile flow [33]. More recently, PAVMs were attributed to the absence of hepatic venous flow to the pulmonary circulation. It was observed that PAVMs develop in patients with cavo-pulmonary connections without a hepatic venous efflux to the pulmonary circulation, e.g. after the Kawashima (bilateral bidirectional cavopulmonary anastomoses for patients with bilateral SVCs) or Glenn procedure without Fontan completion or in the presence of left atrial isomerism with hepatic veins draining directly to the atria and systemic circulation [32, 33]. PAVMs can resolve when hepatic venous blood flow to the lung is reestablished, which has led to the assumption that a, yet unknown, component of the hepatic venous blood, named “hepatic factor”, is critical for sustaining the integrity of the pulmonary vasculature [15]. This “hepatic factor” is believed to inhibit mediators that promote angiogenesis (e.g., VEGF), thus, its exclusion from the pulmonary circulation induces vascular neogenesis [15, 32, 34, 35].

The concept of a hepatic regulator of angiogenesis remains a scientific puzzle. Given the inhibitory function of miRNAs in post-transcriptional gene regulation, the hepatic factor’s proposed role in inhibiting the formation of PAVMs and the elevated VEGF levels in hepatic venous blood, one could hypothesize that the hepatic factor is a miRNA that maintains pulmonary vascular homeostasis by inhibiting VEGF synthesis [34]. However, the potential role of miRNA in the pathogenesis of PAVMs is unclear and warrants further investigation. An alternative hypothesis proposes the involvement of endostatin, a potent inhibitor of VEGF and angiogenesis, in the development of PAVMs [35]. The diversion of hepatic blood flow away from the pulmonary circulation inhibits the production of endostatin, a fragment derived from the C-terminal domain of the α1 chain of the hepatocyte-specific XVIII collagen, potentially by disrupting its first-pass metabolism in the lungs. Thereby, the reduced endostatin levels may favor PAVMs development [35].

### Other factors responsible for pulmonary vascular disease

**Fig. 1:**
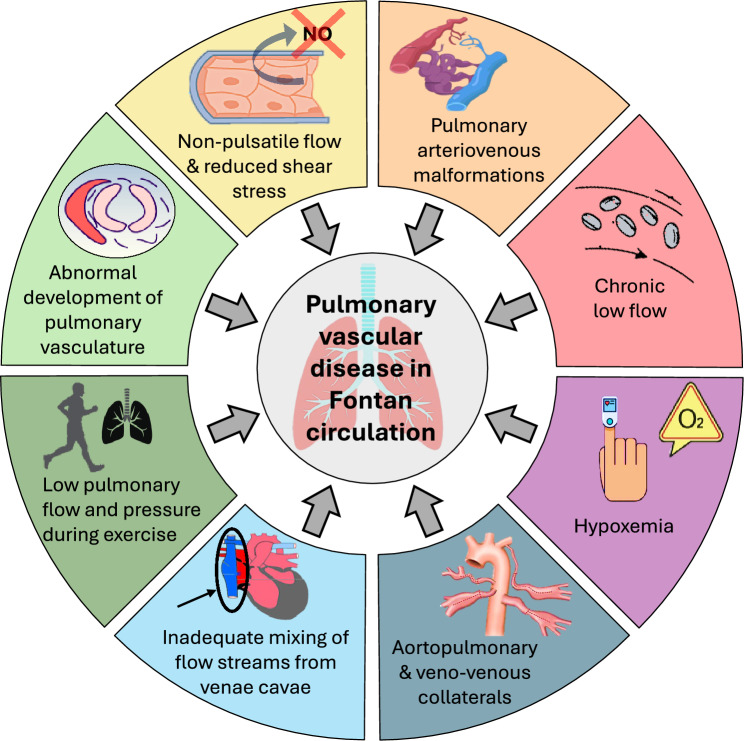
Mechanisms contributing to pulmonary vascular disease in the Fontan circulation

Additional factors potentially contributing to pulmonary endothelial dysfunction in patients with a Fontan circulation include a chronically low pulmonary blood flow, chronic desaturation, increased flow from aortopulmonary and veno-venous collaterals, inadequate mixing of blood streams from the inferior and superior venae cavae, and inability to substantially increase pulmonary flow and pressure during exercise [13] (Fig. 1). Collectively, these factors contribute to a complex hemodynamic profile in these patients, potentially causing a rise in PVR.

## Endothelial dysfunction affecting the systemic circulation in Fontan patients

Profound anatomical alterations following the Fontan procedure precipitate substantial changes in hemodynamics, affecting not only the pulmonary but also the systemic circulation [36]. Indeed, several studies have confirmed the presence of systemic vasculopathy using circulating and vascular biomarkers (Table 1). Although accumulating evidence highlights the complex interplay between the pulmonary and systemic vasculature, systemic vascular involvement in the Fontan physiology has not attracted much attention.

**Table 1:** Summary of endothelial dysfunction biomarkers investigated in Fontan circulation

Biomarker	Fontan population (pediatric/adult)	Main findings in Fontan patients^*^	Significant associations
**NOS inhibitors**			
ADMA	Pediatric	↑ [5] vs published normal values in children	Trend for inverse correlation with FMD [5]
SDMA	Pediatric	↑ [5] vs published normal values in children	Inversely correlated with FMD [5]
**Endothelin-1**	Pediatric & adult	↑ [37–39] ↔ [10]	Positively correlated with PVR [38] and cardiothoracic ratio [40] Predictor of cardiovascular death [40] Higher in Fontan patients with reduced cardiac function [10]
**VEGF**	Pediatric & adult	↑ [41, 42]	Inversely correlated with age [42] Associated with worsening hepatic dysfunction [43]
**Circulating EPCs**	Pediatric	↓ [44]	Inversely correlated with AIx [44]
**Inflammatory markers**			
Galectin-3	Adult	↑ [45]	Positively correlated with age, uric acid and hsCRP [45] Negatively correlated with eGFR [45] Associated with increased risk of death or non-elective cardiovascular hospitalization [45]
hsCRP	Adult	↔ [39, 46]	Elevated hsCRP (≥ 2.98 mg/L) associated with increased risk of death or non-elective cardiovascular hospitalization [47] Associated with cardiothoracic ratio [40]
TNF-a	Pediatric & adult	↑ [39, 44]	Positively correlated with PWV [39]
sTNFR2	Pediatric	↓ [44] vs published normal values	Increased in patients with protein-losing enteropathy [44]
IL6	Adult	↑ [41]	NA
Syndecan-1	Adult	↑ [46]	NA
Glycophorin-A	Adult	↑ [46]	NA
Leukemia inhibitory factor	Adult	↓ [46]	NA
Nerve growth factor-ß	Adult	↓ [46]	NA
**Cell adhesion molecules**			
VCAM, ICAM	Pediatric & adult	↑ [41]	Tadalafil had no significant effect on plasma levels of VCAM and ICAM [48]
**Coagulation pathway molecules**			
*Coagulation factors and inhibitors*			
FII (prothrombin), FV, FVII, FVIII, FIX, FX, Fibrinogen	Pediatric & adult	↓ [49–51] ↓ in pre-Fontan pts [52]	FII, FV, FVII, FVIII and FX positively correlated with SaO_2_ [49, 50] FVII and FV positively correlated with serum prealbumin [51] FVII positively correlated with protein C and antithrombin III [51] FII, FV, FVII and FX negatively correlated with bilirubin level [52] FVIII positively correlated with SVC pressure [50]
Prothrombin fragments F1.2	Adult	↑ [49]	Positively correlated with age [49] Positively correlated with clot lysis time and TAFI activity [49]
Antithrombin III	Pediatric & adult	↑ [49, 51] ↓ in pre-fontan [52] and Fontan [50]	Positively correlated with FVII, serum prealbumin [51] and SaO_2_ [52]
Free TFPI	Adult	↑ [49]	NA
Thrombin-antithrombin complex III	Pediatric & adult	↑ [53]	NA
Protein C	Pediatric & adult	↔ [49, 51] ↓ in pre-Fontan [52] and Fontan [50]	Positively correlated with FVII, serum prealbumin [51] and SaO_2_ [52]
Protein C activity	Pediatric & adult	↓ [53]	NA
Protein S	Pediatric & adult	↓ [49] ↓ in pre-fontan [52]	Positively correlated with SaO_2_ [49, 52] Inversely correlated with sP-selectin [49] Independent predictor of thromboembolic events late after Fontan surgery [49]
*Fibrinolysis variables*			
a2AP	Adult	↔ [49]	NA
Plasminogen	Pediatric & adult	↔ [49] (adults) ↓ [50] (children)	NA
t-PA Ag	Pediatric & adult	↔ [54] at the basal condition	NA
PAI-1 Ag	Pediatric & adult	↔ at the basal condition [54] Significant variable in the multivariate discriminant analysis [54] ↑ [49]	Positively correlated with clot lysis time and TAFI activity [49]
PAI-1 activity	Pediatric & adult	↔ [49, 54]	Positively correlated with TAFI activity [49]
TAFI activity	Adult	↑ [49]	Positively correlated with clot lysis time, PAI-1 activity, PAI-1 antigen and F1.2 [49]
TAFI antigen	Adult	↔ [49]	NA
*Platelet and endothelial activation markers*			
Thrombomodulin	Pediatric & adult	↓ [53] (children) ↔ [49] (adults)	Negatively correlated with the postoperative interval after the Fontan procedure [53]
vWF:Ag		↑ [49, 54]	NA
sP-selectin	Pediatric & adult	↑ [49, 53]	Inversely correlated with SaO_2_, prothrombin, FV, FIII, FIX, FX and free protein S [49]
sCD40L	Adult	↑ [49]	Independent predictor of thromboembolic events late after Fontan surgery [49]
**Neurohumoral markers**			
Norepinephrine	Pediatric & adult	↑ [37, 55, 56]	Positively correlated with PAWP [37] Inversely correlated with LVEF and β^†^ [55]

a2AP, Antiplasmin; ADMA, Asymmetric Dimethylarginine; AIx, augmentation index; BNP, brain natriuretic peptide; CVP, central venous pressure; eGFR, estimated glomerular filtration rate; EPCs, endothelial progenitor cells; FMD, flow-mediated dilatation; HR, heart rate; hsCRP, high sensitivity C-reactive protein; ICAM, intercellular adhesion molecule; IL6, interleukin-6; IVC, inferior vena cava; LVEF, left ventricular ejection fraction; NYHA, New York Heart Association; vWF:Ag, von Willebrand factor antigen; t-PA Ag, tissue-type plasminogen activator antigen; PAI Ag, plasminogen activator inhibitor antigen; PAWP, pulmonary arterial wedge pressure; PVR, pulmonary vascular resistance; PWV, pulse wave velocity; SaO2, arterial oxygen saturation; sCD40L, soluble CD40 ligand; SDMA, symmetric dimethylarginine; sP-selectin, soluble P-selectin; SVC, superior vena cava; TAFI, thrombin activatable fibrinolysis inhibitor; TFPI, free tissue factor pathway inhibitor; TNF-a, tumor necrosis factor-alpha; VCAM, Vascular cell adhesion molecule; VEGF, vascular endothelial growth factor

^*^If no comparator is stated, the comparison is made between Fontan patients and controls; otherwise, the comparator is clearly stated

^†^β: the increase in HR from 1 min after the full-dose atropine infusion to 6 min after the isoproterenol continuous infusion

**Fig. 2:**
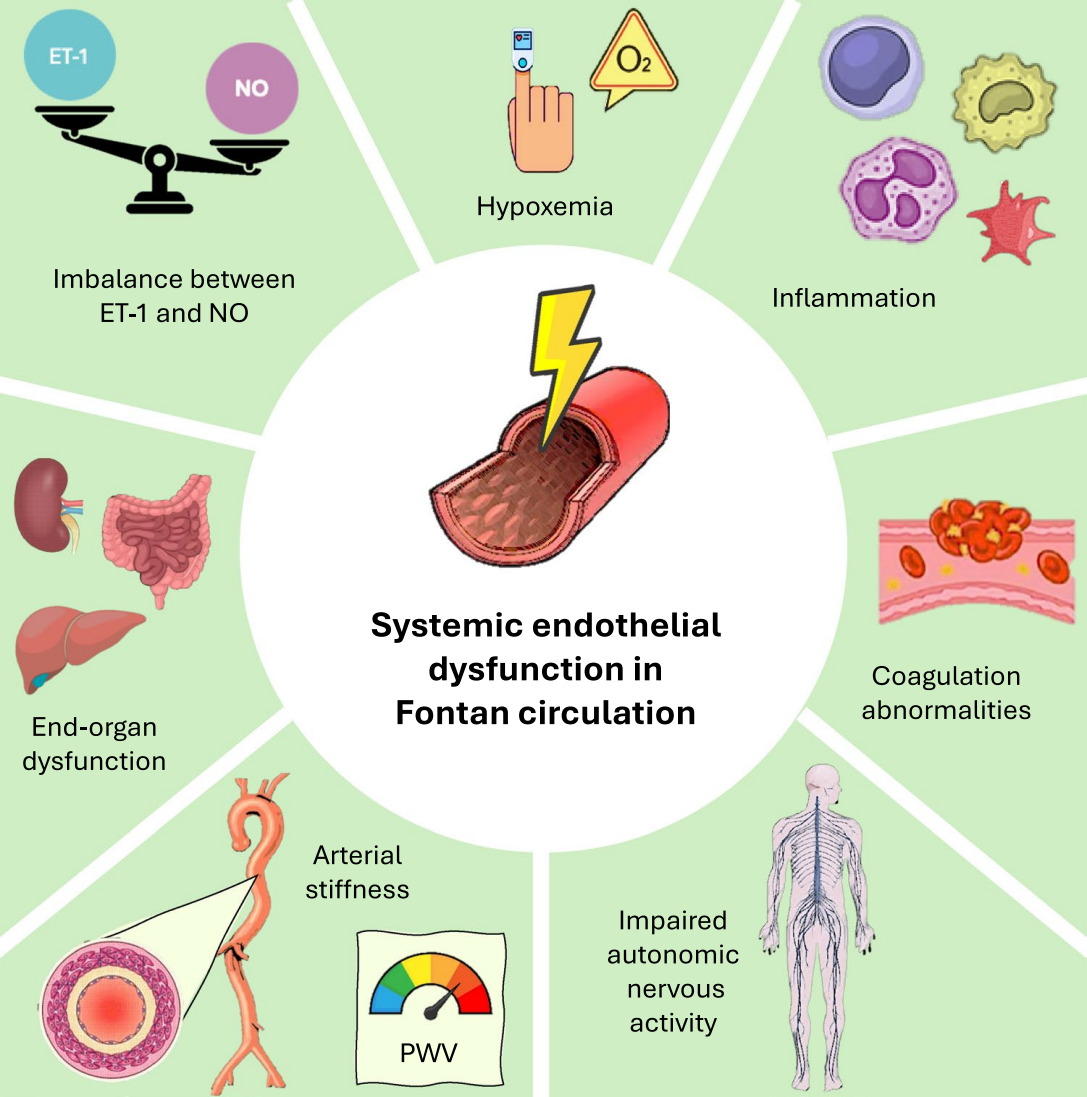
Mechanisms contributing to systemic endothelial dysfunction in the Fontan circulation. Abbreviations: ET-1, endothelin-1; NO, nitric oxide

Several potential pathophysiological mechanisms may contribute to the development of systemic vasculopathy, alongside PVD, in patients with Fontan circulation (Fig. 2). The key mechanisms are outlined and analyzed below.

### Imbalance between ET-1 and NO

Impairment of the NO pathway contributes to the transition of the endothelium into a non-adaptive state, characterized by imbalance between vasodilatory and vasoconstrictive substances, and vascular remodelling [7, 57]. The increased plasma concentrations of asymmetric dimethylarginine (ADMA) and symmetric dimethylarginine (SDMA) in Fontan patients, both of which are endogenous inhibitors of eNOS, support the hypothesis of reduced NO synthesis [5, 18]. Additionally, elevated plasma levels of endothelin-1 (ET-1), a potent endothelium-derived vasoconstrictor, have been observed in Fontan patients and may serve as a compensatory mechanism for the reduced cardiac output following the Fontan procedure [15, 38, 39]. ET-1 may be released in response to elevated shear stress on the endothelium caused by the high central venous pressure required to sustain cardiac output [38]. Interestingly, a correlation has been described between serum ET-1 levels and PVR in a small study of Fontan patients, suggesting that vasoconstriction in the peripheral vascular system is linked to pulmonary vasculopathy [38].

### Chronic hypoxia

**Fig. 3:**
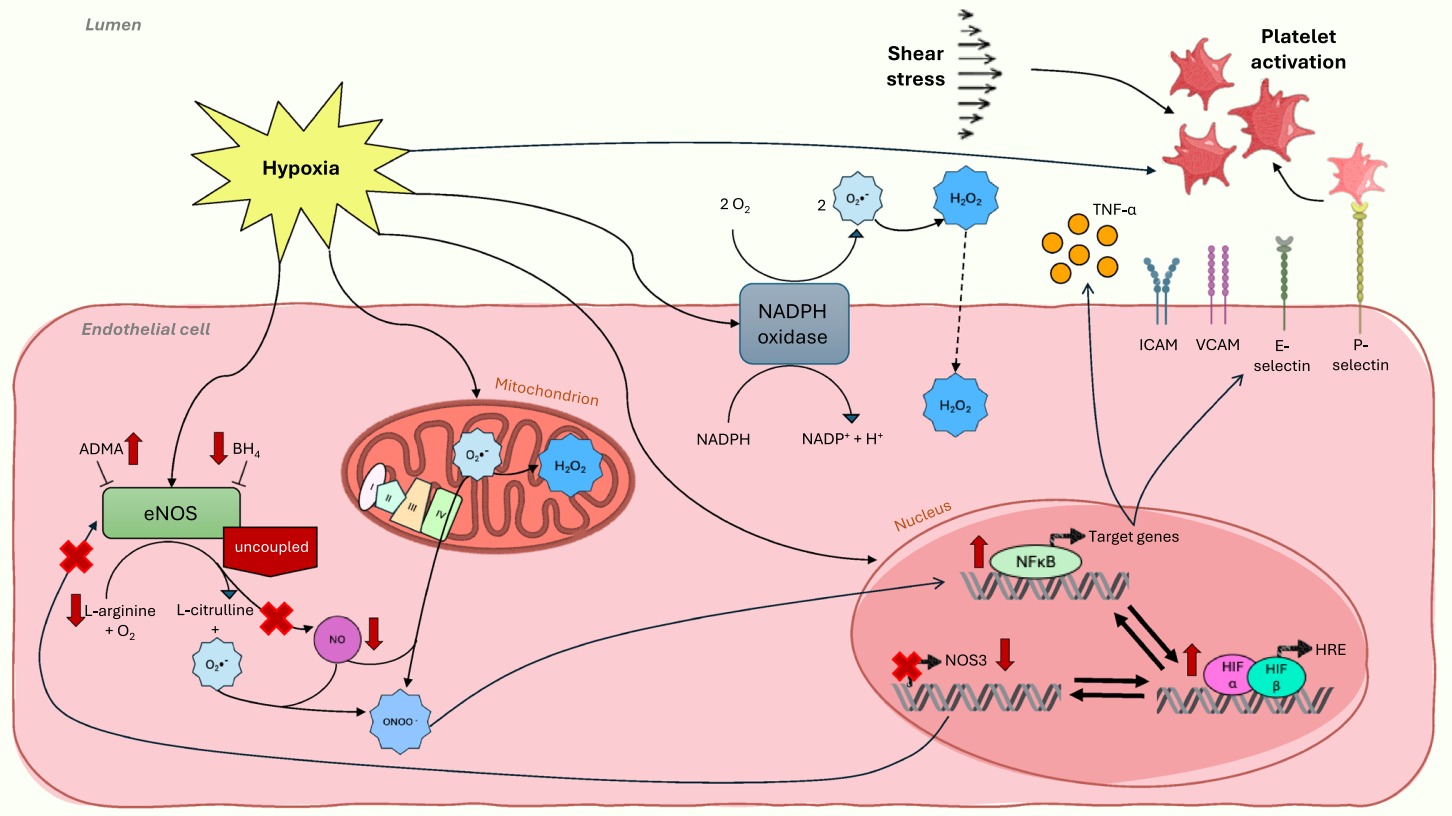
Overview of the interplay between hypoxia, oxidative stress, inflammation and thrombosis in the Fontan circulation. Under hypoxic conditions, deficiency of the substrate L-Arg, accumulation of ADMA, and deficiency of the cofactor BH4 promote eNOS uncoupling, disrupting electron transport from NO synthesis and resulting in the generation of superoxide (O_2_^-^). O_2_^-^ reacts with the remaining NO (produced in reduced amounts) forming peroxynitrite (ONOO⁻), which oxidizes BH4, further exacerbating eNOS uncoupling, and perpetuating a vicious cycle of oxidative stress. Additional sources of hypoxia-induced free radicals arise from the mitochondrial respiratory chain and the NADPH oxidase. Furthermore, hypoxic signaling suppresses eNOS expression, by decreasing the transcription of NOS3 gene and reducing mRNA stability. During hypoxia, HIF-α dimerizes with HIF-β to form a heterodimeric complex that binds to HREs, inducing transcription of NF-κB. Once activated, NF-κB promotes the expression of proinflammatory genes, including cytokines (e.g. TNF-α), and adhesion molecules (e.g. VCAM-1, ICAM-1, and E-selectin). Finally, increased shear stress and hypoxia stimulate platelet activation, promoting thrombosis. Abbreviations: ADMA, Asymmetric dimethylarginine; BH4, tetrahydrobiopterin; eNOS, endothelial nitric oxide synthase; E-selectin, endothelial selectin; HIF, hypoxia-inducible factor; HRE, hypoxia response elements; ICAM, intercellular adhesion molecule; NADPH, nicotinamide adenine dinucleotide phosphate; NF-κB, nuclear factor κB; NO, nitric oxide; P-selectin, platelet selectin; TNF-a, tumor necrosis factor-alpha; VCAM, vascular cell adhesion molecule

Prolonged exposure of patients to chronic hypoxia prior to the Fontan operation is correlated to impaired flow-mediated dilation (FMD), suggesting that the hypoxic environment preceding the Fontan procedure may be a contributing factor to endothelial dysfunction in these patients [58]. Despite the theoretical normalization of systemic oxygen saturations achieved by the Fontan circulation, mild hypoxemia frequently persists, e.g., due to shunting through a surgical fenestration, baffle leaks, PAVMs, or veno-venous collaterals [59]. Moreover, even in normoxemic patients, local tissue hypoxia may still occur as a consequence of low cardiac output and diminished tissue perfusion [41]. Hypoxia drives a phenotypic shift in the systemic endothelium towards a vasoconstrictive state by impairing NO bioavailability [60]. Hypoxic signaling suppresses eNOS expression by decreasing transcription and mRNA stability, and reduces its enzymatic activity via alterations in post-translational modifications [60, 61]. Moreover, hypoxia causes depletion of tetrahydrobiopterin (BH4), an essential cofactor for eNOS, and L-arginine, the substrate for NO generation, hence inducing eNOS uncoupling [60]. This results in the synthesis of superoxide instead of NO, contributing to oxidative stress. Additionally, hypoxia enhances the production of free radicals from the mitochondrial respiratory chain and nicotinamide adenine dinucleotide phosphate (NADPH) oxidase, while it simultaneously promotes endothelial cell activation, and a proinflammatory and procoagulant state [60] (Fig. 3).

At cellular level, these hypoxic responses are mainly mediated by the hypoxia inducible factor (HIF), a key transcription factor induced under hypoxia that regulates the transcription of several genes, including those encoding endothelin-1 and VEGF [60, 62]. During hypoxia, HIF-α dimerizes with the HIF-β subunit to form an active heterodimeric complex that binds to hypoxia response elements (HREs) [60]. This induces the expression of numerous target genes promoting erythropoiesis, angiogenesis, glycolysis and inflammation [60]. Surprisingly, a recent study that investigated proteomic changes in Fontan circulation demonstrated comparable HIF-1α levels in patients and controls. However, this finding could be attributed to the short half-life of HIF-1α and its intracellular release, in parallel with multiple downstream changes; therefore, it does not eliminate the role of HIF in this patient population [41].

VEGF is regarded as another potent mediator of hypoxia-induced vascular remodeling in Fontan circulation. Chronic hypoxia enhances lung tissue expression and serum concentrations of VEGF, which promote aberrant angiogenesis [62–65]. In noncyanotic Fontan patients, however, elevated serum VEGF levels [41–43] may result not just from exposure to hypoxia in early life, but also from the chronically increased systemic venous pressure and mechanical stretching of venous endothelial cells, which induce VEGF production [42]. Furthermore, VEGF plays a pivotal role in the differentiation of endothelial progenitor cells (EPCs), which in turn facilitate vascular repair. The reduced levels of circulating EPCs, despite the VEGF stimulus described in Fontan patients, and their inverse correlation to the augmentation index obtained from peripheral arterial tonometry, suggest their involvement in the pathogenesis of systemic vasculopathy in this population [44].

Secondary erythrocytosis may also contribute to peripheral endothelial dysfunction in cyanotic patients with single ventricle physiology [66]. This adaptive response to chronic hypoxia leads to an increased blood hyperviscosity, thereby increasing vascular shear stress, which theoretically serves as a key stimulus for NO release. Nevertheless, in the setting of cyanosis, this vasodilatory effect is diminished, possibly due to the elevated hemoglobin level, which promotes NO scavenging [67, 68].

### Inflammation

Data linking chronic hypoxemia to increased inflammatory responses suggest a dynamic interplay between these two processes, collectively contributing to endothelial injury [60]. This relationship is likely mediated by HIF activation and increased oxidative stress, which activate the NF-κB transcription factor promoting the expression of proinflammatory genes [60]. The resultant hypoxia-induced inflammatory milieu further compromises vessel wall integrity, driving excessive production of reactive oxygen species (ROS) and reducing NO bioavailability. The consequent vascular remodeling, wall stiffening and lumen narrowing further impair oxygen delivery to tissues, initiating a vicious cycle [60, 69] (Fig. 3). The increased levels of inflammatory markers (e.g., TNF-a, galectin-3, syndecan-1, glycophorin-A) in the Fontan circulation indicate that chronic inflammation, either due to hypoxia or as a possible maladaptive response to chronic low flow, may be involved in endothelial dysfunction [44, 44, 46, 47]. Moreover, the positive correlation of TNF-a with carotid-femoral pulse wave velocity implies that the circulating levels of inflammatory biomarkers may parallel the severity of systemic vasculopathy [39].

This inflammatory cascade, mediated by NF-κΒ activation, drives the overexpression of cell-surface adhesion molecules, including intercellular adhesion molecule (ICAM), vascular cell adhesion molecule (VCAM), and endothelial selectin (E-selectin), which facilitate leukocyte migration into the vascular wall [60] and are among the most endothelium-specific markers of endothelial injury [70]. While increased soluble forms of these adhesion molecules have been observed in other types of congenital heart disease [71], their role in promoting endothelial dysfunction in the Fontan circulation remains inadequately defined.

### Coagulation abnormalities

Vascular injury following the surgical disruption of the endothelium and the implantation of thrombogenic prosthetic materials in the Fontan circuit, impairs critical aspects of endothelial function, such as the regulation of thrombosis and fibrinolysis [72, 73]. Elevated plasma levels of von Willebrand factor (vWF), a protein secreted by activated or dysregulated—but not necessarily damaged—endothelial cells, serve as a marker of endothelial injury in these patients and may suggest a hypercoagulable state, as vWF promotes platelet adhesion, aggregation, and stabilizes coagulation factor VIII in clot formation [49, 54]. Hypercoagulation has also been demonstrated as coagulation factor abnormalities that have been documented both before and after the Fontan procedure. Specifically, significantly lower levels of circulating anticoagulants, including protein C, protein S and antithrombin [49, 50, 52], have been identified compared to controls, along with a paradoxical decrease in coagulation factors (FII, FV, FVII, FVIII, FIX, FX, Fibrinogen) [49–52]. It has, thus, been postulated that Fontan patients exhibit an intricate hemostatic derangement, marked by diminished coagulation factor synthesis, which predisposes them to hemorrhage, juxtaposed with impaired fibrinolysis that promotes thrombosis, ultimately perturbing the hemostatic equilibrium toward a prothrombotic state [49].

Platelet activation, intimately associated with increased shear stress and endothelial dysfunction in the systemic vasculature, could represent an additional mechanism contributing to thrombosis in patients with a Fontan circulation [53]. Elevated levels of sP-selectin and sCD40L support the hypothesis of enhanced platelet activation in Fontan patients [49, 53]. P-selectin, predominantly secreted by activated platelets, is a biomarker of platelet activation and inflammation, while it also promotes coagulation through the upregulation of tissue factor expression and by serving as a catalytic surface for prothrombinase complex formation [49].

The pathophysiology behind these hemostatic abnormalities in Fontan patients may be related to hypoxia, as hypoxic conditions are recognized to drive endothelial dysfunction towards a prothrombotic phenotype. The positive correlation of protein C, protein S and antithrombin with tissue oxygen saturation further underscores the role of hypoxemia in promoting hypercoaguability [52]. However, the presence of endothelial activation or dysfunction in normoxemic Fontan patients suggests the involvement of additional contributing factors, such as low, non-pulsatile flow, chronic venous congestion, hepatic dysfunction and protein-losing enteropathy [54]. The synergistic effects of these conditions, along with mechanical endothelial injury during surgical procedures, may collectively contribute to endothelial perturbations that predispose to thrombosis [54].

### Impaired autonomic nervous activity

Deranged cardiac autonomic nervous activity, evidenced by reduced heart rate variability and baroreflex sensitivity [74], could be another pathway implicated in endothelial dysfunction in the Fontan circulation [55, 74]. The impaired cardiac autonomic nervous control may partially arise from the inevitable surgical disruption of cardiopulmonary nerves surrounding the vessels and atrium [55]. The increased muscle sympathetic nerve activity (MSNA) and plasma norepinephrine concentrations observed in Fontan patients support the presence of sympathetic overactivation in this population, which possibly represents an indispensable compensatory mechanism, essential for preserving systemic blood flow and adequate perfusion of vital organs [10, 56]. This increased sympathetic activity of the peripheral vasculature may be responsible for the increased systemic vascular resistance, potentially possessing a pivotal role in the progressive failure of the Fontan circulation [10]. Coupled with a pronounced reduction in parasympathetic activity, this imbalance may also predispose to arrhythmias and act as a significant contributor to sudden cardiac death [55].

### Arterial stiffness

The systemic vascular endothelium is instrumental in regulating vascular tone and, consequently, arterial elasticity; however, the endothelial mechanisms underlying the adaptation of arterial mechanics to alterations in blood flow remain inadequately characterized [75]. Speculated pathways leading to impaired vasodilation and increased arterial stiffness implicate impaired shear stress-induced endothelial cell hyperpolarization and reduced production of endothelial-derived hyperpolarizing factor (EDHF) and NO in response to agonist stimulation [75].

Recent data have revealed elevated arterial stiffness in Fontan patients compared to controls, with notable differences primarily observed in the ascending aorta and aortic arch, whereas no significant differences have been consistently reported in the descending thoracic aorta, abdominal aorta, or carotid artery [76–78]. In functionally univentricular hearts, increased arterial stiffness is instrumental, as even marginal increases in afterload can expedite the progression of ventricular dysfunction, a primary determinant of Fontan circulation failure [39, 76]. The pathophysiological mechanisms of arterial stiffness are intricate and yet to be defined. Apart from accelerated vascular ageing, chronic cyanosis, volume overload, inflammation, shear or metabolic stresses preceding the surgical corrections [10, 39, 76], additional factors may be involved, such as the presence of homograft or synthetic graft material in patients with a reconstructed aortic arch in the context of hypoplastic left heart syndrome [76].

### End-organ dysfunction

The distinctive pathophysiological features of the Fontan circulation, characterized by chronic venous congestion and diminished cardiac output, predispose patients to a spectrum of end-organ complications, including liver fibrosis, cirrhosis, hepatocellular carcinoma, renal dysfunction, protein-losing enteropathy and plastic bronchitis [13]. This may further exacerbate endothelial dysfunction through diverse mechanisms that ultimately establish a pro-inflammatory, hypoxic state, thereby sustaining a vicious cycle of vascular damage. A mechanistic link between endothelial dysfunction and end-organ damage has, therefore, been proposed; however, the pathophysiological mechanisms underlying this bidirectional relationship remain unclear [79].

## Unresolved issues and future perspectives

Despite the intriguing evidence regarding endothelial dysfunction in the Fontan circulation, substantial gaps remain in fully elucidating the underlying pathophysiological mechanisms and understanding its precise associations with various critical clinical parameters. Identifying such correlations could prove useful in a better understanding of the Fontan (patho-)physiology and in the earlier detection of patients at high risk for developing Fontan failure. Combining proteomics-based with traditional biomarkers represents a promising avenue for individualized risk stratification strategies in Fontan patients, enhancing precision in prognostication and tailored clinical management. However, the incorporation of such biomarkers into prognostic tools for predicting adverse cardiovascular outcomes remains currently far from routine clinical practice.

Existing data predominantly derives from observational studies with relatively small sample sizes and is therefore insufficient for establishing clear cause-and-effect relationships. Future, well-designed research studies, and/or randomized trials targeting specific molecular pathways are essential. By strategically modulating these pathways, such trials can elucidate causal mechanisms and assess the therapeutic potential of addressing molecular derangements leading to the deterioration of Fontan circulation. Since endothelial dysfunction coupled with the low cardiac output, venous congestion, and hypoxia contribute to multiorgan failure and adverse outcomes, identifying modifiable molecular mediators and advancing precision medicine strategies are of paramount importance. Such targeted approaches could mitigate the progression of Fontan-related complications, thereby improving long-term prognosis and enhancing eligibility for transplantation.

Beyond pharmacological treatments, exercise-based rehabilitation is a promising non-pharmacological tool for enhancing endothelial function in cardiovascular diseases by activating eNOS, facilitating endothelial progenitor cells mobilization and inhibiting pro-inflammatory cytokines [80, 81]. While exercise training has demonstrated significant benefits in Fontan circulation, with studies showing that lower limb resistance training generates pulmonary pulsatile flow and enhances peripheral muscle mass, venous return, cardiac output and peak oxygen consumption, and that inspiratory muscle training improves resting cardiac output and exercise ventilatory efficiency [13], its efficacy in reversing endothelial dysfunction remains to date uncertain. To address this, an ongoing randomized controlled trial in pediatric Fontan patients is currently evaluating whether a combined aerobic and resistance exercise intervention can improve endothelial function [82]. The results of such studies are highly anticipated to provide further insights into the therapeutic potential of exercise rehabilitation in reversing or improving endothelial dysfunction.

## Conclusions

Patients with Fontan circulation demonstrate endothelial dysfunction of multifactorial etiology that involves both the pulmonary and peripheral vasculature. The underlying pathophysiological mechanisms may affect the pulmonary and systemic circulation differently yet appear interrelated and cumulatively contribute to progressive end-organ failure, ultimately resulting in high morbidity and mortality. Research should be directed towards understanding the mechanisms of endothelial dysfunction in Fontan circulation and identifying targets for effective pharmacological treatments designed to modify these pathogenic pathways and, more importantly, delay disease progression and mitigate future adverse events.

## Data Availability

No datasets were generated or analysed during the current study.

## Abbreviations

ADMA: Asymmetric dimethylarginine
BH4: Tetrahydrobiopterin
CVP: Central venous pressure
EDHF: Endothelial-derived hyperpolarizing factor
eNOS: Endothelial nitric oxide synthase
EPCs: Endothelial progenitor cells
E-selectin: Endothelial selectin
ET-1: Endothelin-1
FMD: Flow-mediated dilatation
ICAM: Intercellular adhesion molecule
MSNA: Muscle sympathetic nerve activity
NADPH: Nicotinamide adenine dinucleotide phosphate
NF-κB: Nuclear factor κB
NO: Nitric oxide
PAVMs: Pulmonary arteriovenous malformations
PVD: Pulmonary vascular disease
PVR: Pulmonary vascular resistance
ROS: Reactive oxygen species
SDMA: Symmetric dimethylarginine
VCAM: Vascular cell adhesion molecule
VEGF: Vascular endothelial growth factor
vWF: Von Willebrand factor
